# Mitochondria: An Organelle of Bacterial Origin Controlling Inflammation

**DOI:** 10.3389/fimmu.2018.00536

**Published:** 2018-04-19

**Authors:** Alain Meyer, Gilles Laverny, Livio Bernardi, Anne Laure Charles, Ghada Alsaleh, Julien Pottecher, Jean Sibilia, Bernard Geny

**Affiliations:** ^1^Institut de Physiologie EA 3072, Service de physiologie et d’Explorations Fonctionnelles, Hôpitaux Universitaires de Strasbourg, Strasbourg, France; ^2^Centre de Référence des Maladies Autoimmunes Rares, Hôpitaux Universitaires de Strasbourg, Strasbourg, France; ^3^Fédération de Médecine Translationnelle de Strasbourg, Université de Strasbourg, Strasbourg, France; ^4^Institut de Génétique et de Biologie Moleculaire et Cellulaire, UMR 7104, INSERM U1248, University of Strasbourg, Illkirch, France; ^5^Kennedy Institute of Rheumatology (KIR), University of Oxford, Oxford, United Kingdom; ^6^Pôle d’Anesthésie-Réanimation SAMU-SMUR, Service d’Anesthésie-Réanimation Chirurgicale, Hôpital de Hautepierre, Hôpitaux Universitaires de Strasbourg, Strasbourg, France

**Keywords:** inflammation, mitochondria, reactive oxygen species, myositis, dermatomyositis, rheumatoid arthritis, systemic lupus erythematosus

## Abstract

Inflammation is a cellular and molecular response to infection and/or tissues injury. While a suited inflammatory response in intensity and time allows for killing pathogens, clearing necrotic tissue, and healing injury; an excessive inflammatory response drives various diseases in which inflammation and tissues damages/stress self-sustain each other. Microbes have been poorly implied in non-resolving inflammation, emphasizing the importance of endogenous regulation of inflammation. Mitochondria have been historically identified as the main source of cellular energy, by coupling the oxidation of fatty acids and pyruvate with the production of high amount of adenosine triphosphate by the electron transport chain. Mitochondria are also the main source of reactive oxygen species. Interestingly, research in the last decade has highlighted that since its integration in eukaryote cells, this organelle of bacterial origin has not only been tolerated by immunity, but has also been placed as a central regulator of cell defense. In intact cells, mitochondria regulate cell responses to critical innate immune receptors engagement. Downstream intracellular signaling pathways interact with mitochondrial proteins and are tuned by mitochondrial functioning. Moreover, upon cell stress or damages, mitochondrial components are released into the cytoplasm or the extra cellular milieu, where they act as danger signals when recognized by innate immune receptors. Finally, by regulating the energetic state of immunological synapse between dendritic cells and lymphocytes, mitochondria regulate the inflammation fate toward immunotolerance or immunogenicity. As dysregulations of these processes have been recently involved in various diseases, the identification of the underlying mechanisms might open new avenues to modulate inflammation.

## Introduction

### Inflammation: A Physiological Process of Microbial Clearance and Wound Healing, Whose Deregulation of Intensity, and/or Duration Leads to a Wide Spectrum of Diseases

Inflammation is a cellular and molecular response to infection and/or tissue injuries. During infection, inflammation is typically triggered by exogenous ligands harbored by microbes known as pathogen-associated molecule patterns (PAMPs), that are selective to a given microbial class. In contrast, during tissue injuries, inflammation is triggered by endogenous molecules, normally hidden from the immune system, but actively secreted into the cytosol or passively released into the extracellular milieu upon cell stress or damage. These substances are named damage-associated molecular patterns (DAMPs).

Both PAMPs and DAMPs are sensed by pattern recognition receptors (PRRs), germline-encoded sensors expressed by immune cells [i.e., dendritic cells (DCs), monocytes, polynuclear cells, leukocytes], as well as by resident cells in tissues (epithelium, mesenchyme). The ligation of PRRs stimulates intracellular signaling cascades leading to the expression and secretion of numerous proinflammatory mediators, whose amounts and nature are selective of both the PRR and the cells which harbor them. These factors include vasoactive molecules, chemokines, cytokines, and proteolytic enzymes, that drive vasodilatation, blood immune cell recruitment, and activation (1).

While an inflammatory response adapted in intensity and time allows for killing pathogens, clearing necrotic tissue and healing injury; an excessive response drives various diseases. Exaggerate inflammation intensity notably leads to septic shock (in response to infection) or systemic inflammatory response syndrome (SIRS, in response to posttraumatic tissues damages) during which inflammation itself drives morbidity and mortality. If persistent, both inflammation and tissue damages/stress fuel a feed-forward loop that contributes to numerous pathological conditions (e.g., autoimmune diseases, diabetes, chronic obstructive pulmonary disease, atherosclerosis, aging, etc.). Interestingly, microbes have been poorly implied in excessive inflammation highlighting the importance of understanding the role of endogenous regulation of inflammation (2).

### Mitochondria: From Bacteria to a Regulator of Inflammation

Mitochondria have been historically identified as the main source of cellular energy by coupling the oxidation of fatty acids and pyruvate to the production of high amount of adenosine triphosphate (ATP) by the electron transport chain (ETC). Mitochondria are also the main source of reactive oxygen species (ROS) (3) produced by the reaction between oxygen and a small proportion of electrons (0.15 and 0.8%) that leak mainly from complexes I and III of the ETC (4). These highly reactive molecules act as physiological signals that contribute to various cellular functions (5). However, when produced in excess, ROS react in an uncontrolled manner with proteins, lipids, and DNA, leading to cell dysfunctions and/or death. Mitochondria are an important source of ROS (mtROS), especially when malfunctioning, but are also vulnerable to oxidative damages that can further increase mtROS production (6). Thus, regulation of mitochondria is crucial for cells integrity.

Mitochondrial functions, quality and amount are notably regulated by mitochondrial dynamics and mitophagy. Mitochondrial fusion maximizes oxidative phosphorylation efficiency, and under stressful conditions, mixes the contents of partially damaged mitochondria as a form of complementation. Mitochondrial fission is required to create new mitochondria and to segregate damaged parts of mitochondria that will be eliminated by autophagy (7). The specific removal of damaged mitochondria by autophagy is a regulated process, also called mitophagy, that is critical for preventing the cytotoxic effects induced by mitochondria dysfunctions, and for the maintenance of cellular homeostasis.

Mitochondria’s origin is believed to go back to an endosymbiose of an α-protobacterium in the ancestry of eukaryotic cell that happened about 1.5 billion years ago (8). Surprisingly, this organelle of a bacterial origin has since then, not only been tolerated by immunity, but has also been placed as a central regulator of cell danger responses, acting at all steps of inflammatory responses. Mitochondria contribute to PRRs responses, as they represent a check point of the intracellular cascades downstream numerous PRRs. In addition, mitochondria trigger inflammation by acting as DAMPS and polarize the fate of inflammatory responses by regulating the energetic level of immune cells.

## Mitochondrial Regulation of Innate Immunity

### Mitochondria Are a Platform for PRRs Signal Transduction and Regulation

**Figure 1:**
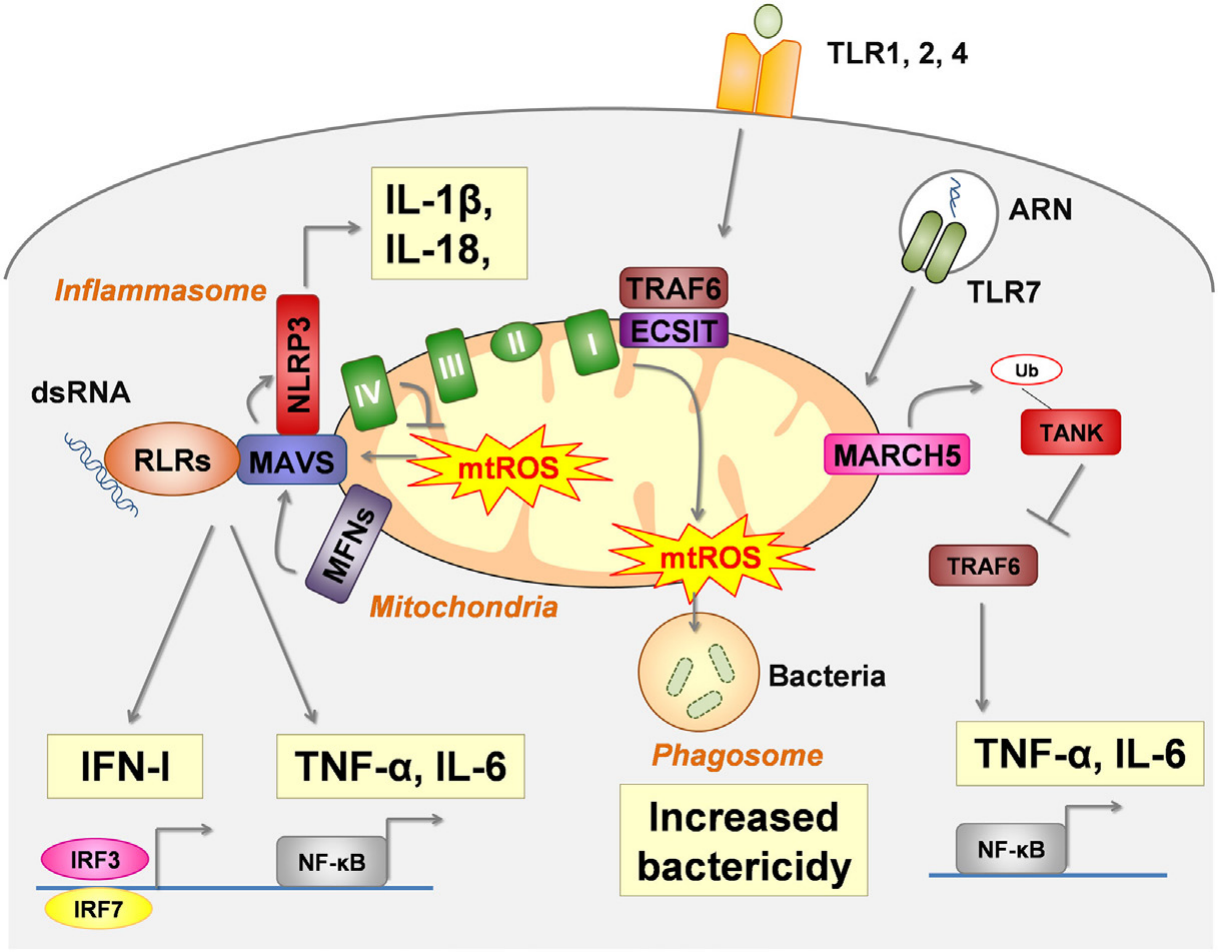
Mitochondria are a platform for pattern recognition receptors (PRRs) signal transduction and regulation. In intact cells, intracellular signaling pathways of several critical PRRs physically interact with mitochondria to act as a modulator. RIG-I-like receptors (RLRs) (retinoic acid- inducible gene I and melanoma differentiation-associated protein 5 MDA5) signals are dependent of MAVS, the activation of which (oligomerization) is enhanced by mtROS and mitochondrial fusion [regulated by mitofusin (MFN) 1 and MFN2]. NLRP3-inflammasome assembly also interacts with MAVS for proper activation. Moreover, mtROS and several mtDAMPs activate the NLRP3-inflammasome (see Figure 2). Toll-like receptor (TLR) 1, TLR2, and TLR4 ligation leads to the enrichment of TRAF6 and Evolutionarily Conserved Signaling Intermediate In Toll Pathway (ECSIT) (a complex I–assembly factor) at the mitochondrial periphery and subsequent recruitment of mitochondria to macrophage phagosomes were they produce mtROS. TLR7 signaling involves the mitochondrial protein membrane-associated ring-CH-type finger 5 (MARCH5), which catalyzes the polyubiquitination and degradation of TANK a repressor of TLR7 signaling pathway.

In intact cells, mitochondria modulate the cellular response to several PRRs engagement by interacting with their downstream intracellular signaling pathways (Figure 1).

#### Retinoic Acid- Inducible Gene I (RIG-I)-Like Receptors (RLRs)

Retinoic acid-inducible gene I (RIG-I)-like receptors are cytosolic sensors that recognize viral double-stranded RNA (dsRNA). Among them, RIG-I recognizes relatively short dsRNA (<1 kbp) with a 5′ tri- or diphosphate group, whereas melanoma differentiation-associated protein 5 (MDA5) binds to long dsRNA (>1 kbp) with or without a 5′ phosphate group (9).

Stimulated-RLRs bind to the mitochondrial antiviral signaling protein (MAVS) at the outer mitochondrial membrane. Following this interaction, MAVS oligomerizes to recruit and stimulate signaling proteins leading to the activation of IRF3, IRF7 and nuclear factor kappa B (NF-κB) and the synthesis of antiviral molecules (IFN-I, IFN-III, IFN-stimulated genes, and proinflammatory cytokines). Both the expression and localization at the outer mitochondrial membrane of MAVS are required for proper antiviral signaling through RLRs (10). In addition, MAVS interacts with mitochondrial proteins, notably the complex IV subunit COX5B (11), and with proteins enhancing mitochondrial fusion, such as MFN1 and 2 (12–15).

Several mitochondrial modifications enhance mtROS production and overdrive RLR signals. *In vitro*, inhibition of mitochondrial complex I (16, 17) and of complex III (11, 16), as well as deletion of COX5B (11) and mitophagy inhibition (17, 18), increase RLRs signaling in a MAVS-dependent manner. In those experiments, ROS were required (11, 17) and sufficient for RLR activation (17). These observations are consistent with a model in which MAVS acts as a channel that positively links RLR signaling and mtROS production. Moreover, it has been suggested that modulation of outer membrane biophysical properties by mtROS is required to MAVS oligomerization (16).

Mitochondrial dynamics are also implicated in MAVS signaling. Following RLR ligation, mitochondrial fusion, regulated by MNF1 and MNF2, reorganizes MAVS into speckled. Such small aggregates are required (13, 14), but not sufficient (14) for RLRs signaling. Although the exact role of MNF1 and MNF2 on RLR signaling remains unclear (12–15), MFN1 is not required when MAVS is overexpressed (14), indicating that mitochondrial fusion acts upstream of MAVS to promote its efficient oligomerization and downstream signaling.

As IFN-I is in turn able to increase mtROS production (19, 20), deregulation of the connection between mitochondrial function and IFN-I has been recently proposed to play a role in the maintenance of autoimmune diseases. In systemic lupus erythematosus (SLE), both T cells (21) and mesenchymatous stem cells (22) secrete high levels of IFN-I that are associated with mitochondrial dysfunctions, including enhanced mtROS production, and that are reduced by MAVS silencing (22) and ROS inhibition (21). In dermatomyositis, we recently reported that perifascicular muscle fibers, which specifically expressed RIG-1 and IFN-1 stimulated genes, produce high amount of mtROS. In addition, we have shown that ROS scavenging prevents IFN-I stimulated genes in a mouse model of inflammatory myopathy (20).

#### Cyclic GMP-AMP Synthase (cGAS)—Stimulator of Interferon Genes (STING)

Cyclic GMP-AMP synthase is a cytosolic DNA sensor. When ligated to DNA, cGAS induces the synthesis of cyclic GMP-AMP that acts as a soluble second messenger for STING. These events result in a broad stimulation of downstream signals. In opposite to the signaling cascade of other PRRs based on protein–protein interactions, GMP–AMP diffuses *via* gap junctions, transporting the signal between cells (23).

Stimulator of interferon genes is an endoplasmic reticulum (ER) protein associated with MAVS at the mitochondria-associated ER membrane. Once activated, STING translocates from the ER *via* the Golgi to perinuclear endosomes (24, 25), where it activates IRF3, NF-κB, and subsequent interferons and inflammatory cytokines, such as TNF and IL-6. The role of mitochondrial localization of STING remains unclear. It may facilitate RIG-1 signaling, because STING deletion decreases RIG1 pathway (24). It may facilitate its activation by cGAS because, as explained below, cGAS can recognize mitochondrial DNA (mtDNA) extruded in cytoplasm. It may also protect the cell from exaggerated inflammation as the ring finger protein 5 (RNF5) downregulates virus-triggered signaling by targeting STING for ubiquitination and degradation at the mitochondria (26).

#### Toll-Like Receptors (TLR)

Toll-like receptors are a family of transmembrane PRRs that share a similar structure including (i) leucine-rich repeats that mediate the recognition of PAMPs and DAMPs, (ii) a transmembrane domain, and (iii) an intracellular Toll–interleukin 1 receptor domain required for downstream signal transduction. In human, 9 TLRs (TLR1-9), that recognized different PAMPs/DAMPs, have been identified. Upon stimulation, they induce NF-κB, MAPK, IRF3, and/or IRF7 activations and subsequent IFN-I and other proinflammatory cytokines synthesis (27, 28) through MYD88 and/or TRIF intracellular signaling cascades.

In macrophages, the cell surface TLR1, TLR2, and TLR4, that recognize tri-acylated lipopeptides from Gram-negative bacteria and mycoplasma (TLR1 and TLR2 heterodimer) as well as LPS (TLR4), enhance ROS production in the phagosome (known as the macrophage oxidative burst) that is necessary for bactericidal activity. Mitochondria, *via* its respiratory chain, contribute to TLR signaling cascades. TLR1, TLR2, and TLR4 ligation induce the recruitment of mitochondria to macrophage phagosomes, where they produce mtROS. TRAF6, a protein involved in the signal transduction of TLR1, TLR2, and TLR4, translocates to the mitochondria and leads to the enrichment at the mitochondrial periphery of the Evolutionarily Conserved Signaling Intermediate In Toll Pathway (ECSIT), a complex I–assembly factor. ECSIT- and TRAF6-deficient macrophages produce low levels of TLR-induced RLO production, and thus have an altered bactericidal potency (29). ECSIT-induced mtROS production is negatively regulated by peroxyredoxin 6, whose mitochondrial localization is regulated by TLR stimulation at the macrophage surface and control excess of ROS (30).

PBMC isolated from patients with tumor necrosis factor receptor-associated periodic syndrome (TRAPS) produce excess of TNF-α and IL-6 in response to TLR4 engagement (31). This is mediated by a sustained phosphorylation of JNK and p38, that is maintained by ROS from dysfunctioning mitochondria (32). Indeed, ROS inactivate MAPK phosphatases and perpetuate MAPK activation (33).

Toll-like receptor 7 recognizes single-stranded RNA. TLR7 signaling in macrophage is upregulated by the membrane-associated ring-CH-type finger 5 (MARCH5), an ubiquitin ligase of the mitochondrial outer membrane that plays a role in the control of mitochondrial morphology. In response to TLR7 engagement, MARCH5 catalyzes the polyubiquitination and degradation of TANK, a repressor of the TLR signaling pathway. Ablation of MARCH5, or impairment of its mitochondrial outer membrane localization, reduces TLR7-induced NF-κB activation and subsequent IL-6 and TNF-α secretion in macrophages (34).

#### NLRP3-Inflammasome

Inflammasomes are large intracellular protein complexes that ultimately lead to caspase-1 activation and subsequent maturation of the inflammatory cytokines IL-1β and IL-18. The well-described NLRP3-inflammasome activation is a two-step process. First, the expression of inflammasome-related components is induced, including inactive NLRP3, proIL-1β, and proIL-18. This implies activation of the transcription factor NF-κB in response to TLRs stimulation (priming signal). The second step requires the assembly of NLRP3-inflammasomme [including NLRP3, apoptosis-associated speck-like protein (ASC) and procaspase-1] that induces the maturation of procaspase-1 to caspase-1, which cleaves pro-IL-1β and pro-IL-18 into mature cytokines. This second step is controlled by various signals including efflux (such as K + efflux through the purogenic P2X7-receptor), crystals (such as urate, silica, asbestos, amyloid-β, and alum) and mitochondrial components (as discussed below) (35).

NLRP3 inflammasome physically interacts with mitochondria for its assembly. After stimulation, NLRP3 is redistributed from the ER to the perinuclear region (36). In addition, microtubules mediate a dynein-dependent transport of mitochondria to the same region (37), and such colocalization is required for IL-1β secretion (37). Similarly to mitochondrial RLRs regulation, the mitochondrial outer membrane MAVS is required for optimal NLRP3 inflammasome activity *in vitro* and, accordingly, MAVS-deficient mice are protected against acute tubular necrosis along with decreased IL-1β and neutrophils in kidneys (38).

Colocalization of mitochondria and inflammasomes in perinuclear regions is ideal for the inflammasome modulation by mitochondrial components. mtROS are short-lived molecules that act as a signal only in the vicinity of their production. mtROS activate the inflammasome, while mitochondrial clearance by autophagy decreases inflammasome signaling *in vitro* (36). Genetically engineered mice lacking autophagy pathways in macrophages exhibit higher IL-1β levels, neutrophil infiltration, and tissue damages than wild-type mice during sterile experimental peritonitis and fulminant hepatitis (39), as well as higher IL-1β serum levels and increased mortality in septic shock models (40). Accordingly, IL-1β and tubular damages in albumin-overload-induced renal disease in mice were prevented by increasing mitochondrial anti-oxidant defenses (through SOD 2 mimicker) (41). In the same line, human monocytes stimulated by LPS exhibit an anti-oxidant response that prevents rapid maturation of pro-IL-1β. Monocytes from patients with cryopyrin-associated periodic syndromes (a group of autoinflammatory diseases in which NLRP3 gene mutations lead to increased IL-1β secretion), have mitochondrial dysfunctions, increased ROS production and exhausted antioxidant defenses that accelerate the maturation of pro-IL-1β in response to LPS (42, 43). In addition, as discussed below, several mitochondrial DAMPs, such as mtDNA and cardiolipin, activate the inflammasome. Thus, altogether, the current data suggest a model in which inflammasome and mitochondria physically interact to facilitate the modulation of inflammasome by the mtROS and mitochondrial DAMPs.

### Mitochondrial Components Act As DAMPS in Stressed and/or Damaged Cells

**Figure 2:**
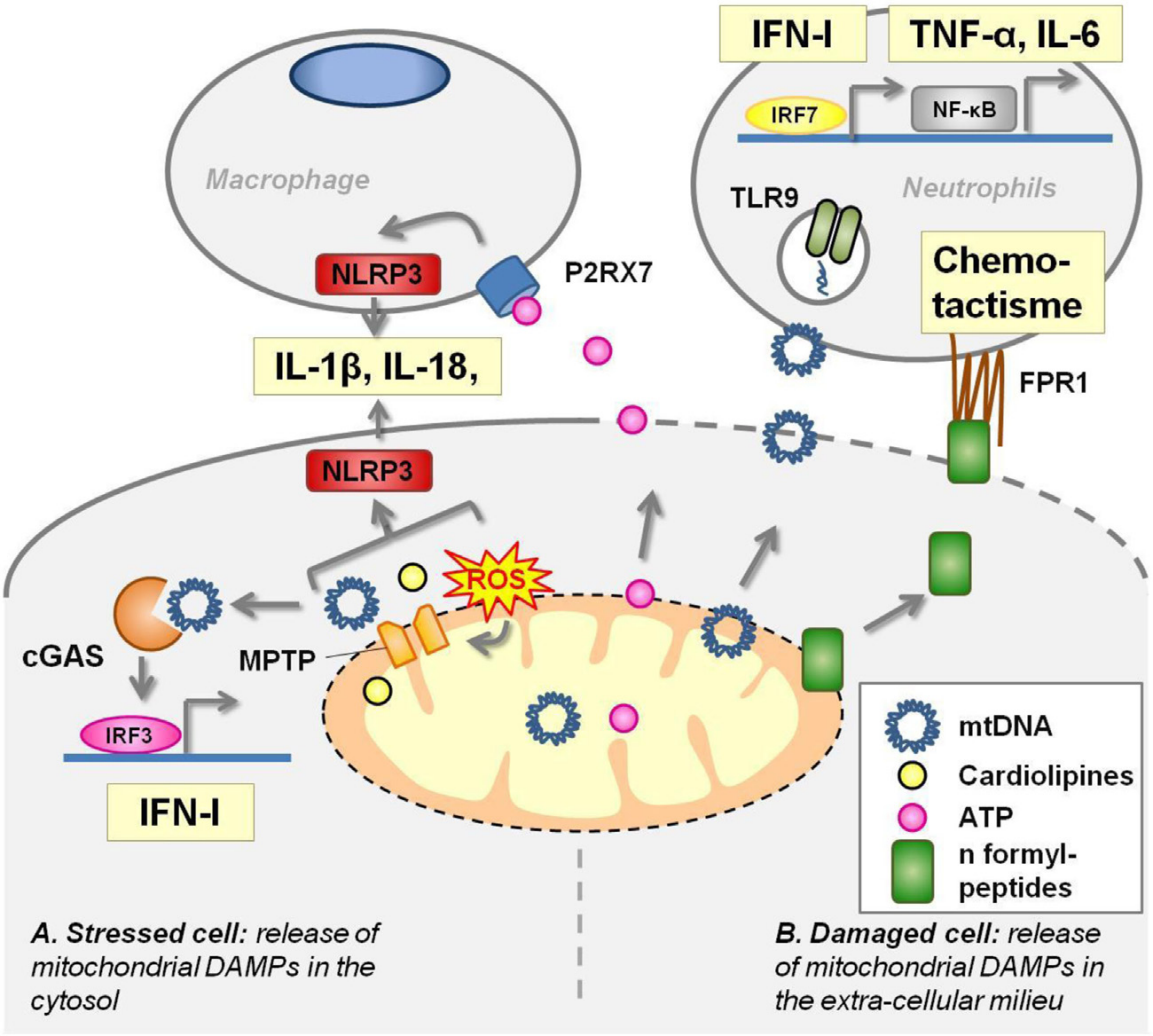
Mitochondrial components act as damage-associated molecular patterns (DAMPS). **(A)** In stressed cells [e.g., viral infection, defect of autophagy, toll-like receptors (TLRs) ligation], mitochondrial transition pore (MPTP) opening extrudes several mitochondrial components in the cytoplasm where they activate pattern recognition receptors (PRRs). Mitochondrial DNA (mtDNA) activates cyclic GMP–AMP synthase (cGAS) receptor and NLRP3 inflammasome. Cardiolipin and mtROS trigger NLRP3 inflammasome. **(B)** Damaged cells release mitochondrial DAMPs into the extracellular milieu where they stimulate PRRs harbored by macrophages and neutrophils. Adenosine triphosphate (ATP) leads to P2X7 opening at the macrophage membrane and triggers NLRP3 inflammasome. mtDNA unmethylated CpG motifs are recognized by TLR9. N-formylmethionine residues in mitochondrial proteins are sensed by the formyl-peptide receptors expressed by neutrophils, driving chemotactism and activation of these cells.

Mitochondria share several molecular features with bacteria that are the hallmark of their microbial origins. In physiological condition, these components do not interact with PRRs. In pathological situations, such as cell damages or stress, they are released in the extra cellular milieu or in the cytoplasm where they act as DAMPs (Figure 2).

#### Mitochondrial DNA

Mitochondrial DNA is a ligand of several innate immune receptors and contributes to the intensity of the inflammation during the response to pathogens, tissue damages, and cell death.

Circular mtDNA has conserved unmethylated CpG motifs, characteristic of bacterial DNA, that are recognized by TLR9 and drive aberrant inflammation in two distinct inflammatory diseases. mtDNA blood levels are markedly increased after a major trauma in patients suffering from SIRS, and venous injection of these mitochondrial DAMPs in rats induces inflammatory lung injuries (44). mtDNA fragments are also present in synovial fluid of rheumatoid arthritis patients, and their amount positively correlates with the severity of the disease (45). Injection of purified mtDNA, but not nuclear DNA, into the joints of mice induces arthritis (dependently on monocyte and granular cells) (46). *In vitro*, mtDNA activates polynuclear neutrophils to the same extent as conventional TLR9 agonists (44, 45).

Upon fibroblast infection with a double stranded DNA virus, mtDNA is released into the cytosol and acts as a ligand of the cytosolic cGAS receptor to potentiate the IFN-1 response and to confer viral resistance (47). Thus, through this mechanism, mtDNA, normally tolerated in the mitochondrial matrix, is used by the cell to boost antiviral responses. Interestingly, such mechanisms also provide an internal control to determine whether mitochondria initiate an immunologically silent or a proinflammatory cell death. Apoptosis is an immunologically silent cell death. In contrast, failure of apoptosis due to caspase 9 deficiency trigger IFN-Is sec-retion through mtDNA activation of cGAS resulting from mitochondrial outer membrane permeabilization by Bax and Bak (48). Similarly, an accelerated and non-apoptotic cell death of neutrophils has been shown to enhance IFN-I production in SLE through cGAS pathway. Exposure to low concentrations of anti-RNP IgG (a characteristic autoantibody of SLE) switches SLE neutrophil death from apoptosis to NETosis, a unique form of death characterized by nuclear disruption and release of neutrophil extracellular traps (NETs). These NETs from SLE patients, and not from healthy donors, are highly enriched in oxidized mtDNA which promotes the secretion of IFN-I by PBMC *in vitro* and in mice spleen through the cGAS pathway (49).

Oxidized mtDNA extrusion in the cytosol through ROS-dependent opening of the mitochondrial transition pore (MPTP) is also required to coactivate the NLRP3 inflammasome. Indeed, macrophages depleted in mtDNA, or treated by mitochondrial ROS scavengers or by cyclosporine (an inhibitor of MPTP), fail to produce IL-1β and IL-18 in response to NLRP3-inflammasome activation by LPS and ATP (40, 50). Moreover, autophagy, which clears mitochondrial ROS, prevents mtDNA extrusion in the cytoplasm and subsequent activation of the inflammasome (40).

#### Adenosine Triphosphate

Mitochondria produce a high amount of ATP that is transported into the cytosol and serve as the main source of cellular energy. In normal condition, ATP is not secreted and furthermore, extracellular ATP is rapidly degraded by exonucleases. Thus, the detection of high amounts of ATP in the extracellular milieu is an efficient way to alert the immune system in case of cell injuries (51). This is ensured by P2X7, an ATP gated channel receptor harbored by macrophages, whose engagement activates NLRP3 inflammasome (step 2) and IL-1β secretion. The marked influx of neutrophils into the peritoneal cavity induced by IP-injected necrotic cells to wild-type mice is dependent on the NLRP3 inflammasome (52). In addition, NLRP3 inflammasome-deficient mice are protected from lethal kidney ischemia reperfusion (52). In a mouse model of focal hepatic necrosis, ATP, its receptor P2X7, NLRP3 activation and subsequent IL-1β production in resident liver macrophages, enhance ICAM-1 expression in endothelial cells that leads to polynuclear recruitment in the injured tissues (53). *In vitro*, mitochondria, but not cytosolic or nuclear particles, nor plasma membrane, from necrotic cells activate the inflammasome in macrophages, and such activation is partially dependent on ATP production by mitochondria and on P2X7 expression (52).

#### Cardiolipin

Cardiolipin is a non-bilayer forming phospholipid that, in eukaryotic cells, is exclusively found in the inner mitochondrial membrane. Its structure is unique as it consists of two diacylated phosphatidyl groups joined by a glycerol bridge (54). In macrophages primed with LPS, cardiolipin and NLRP3 interaction acts as a sufficient and necessary signal for inflammasome activation that is ROS-independent, but MPTP opening dependent (55).

#### Formyl-Peptides

As in bacteria, mitochondria initiate protein synthesis with a N-formylmethionine residue. This molecular pattern is sensed by formyl-peptide receptors (FPRs) that are expressed by neutrophils driving their chemotactism and activation. In a murine model of focal hepatic necrosis, confocal microscopy experiments have shown that mitochondrial formyl-peptide signals released from necrotic cells guide neutrophils through non-perfused sinusoids into the injury (53). *In vitro*, human mitochondrial formylpeptides promote chemotactism, metalloprotease secretion, and oxidative burst of neutrophils (44, 53, 56).

## Mitochondrial Metabolism in DCs and Lymphocytes Regulate the Inflammation Fate

When the innate immune response fails to clear DAMPs and/or PAMPs, it activates and polarizes the adaptive immune response, a set of cellular and molecular defenses, proper to vertebrates, mediated by T cells and B cells, against specific antigens (57). The initiation of adaptive immune response starts with the formation of an immune synapse between DCs, specialized in antigens presentation through class II MHC complexes, and T helper (Th) cells, the orchestrators of the adaptive immune response. Cells metabolic state of the immune synapse has been recently shown to regulate the polarization of the adaptive immune response. Schematically, shifting to glycolytic metabolism drives a proinflammatory response while shifting to oxidative metabolism drives an anti-inflammatory response.

Under the control of mitochondrial respiration, DC induce pro- or anti-inflammatory differentiation of Th cells depending on the expression of costimulatory molecules or of check point inhibitor ligands on their membrane (58). In DCs, TLR-induced costimulatory molecules expression is dependent on reduced mitochondrial respiration, associated with increased glycolysis. Increasing DC-mitochondrial respiration by AICAR treatment antagonizes TLR effects (59). Accordingly, peroxisome proliferator-activated receptor γ, a member of the nuclear receptor superfamily, is a potent inducer of mitochondrial biogenesis, a negative regulator of DC costimulatory molecules, and drives anergy of Th (60).

Moreover, mitochondrial respiration controls the subsequent activation and differentiation of Th cells, as it requires mitochondrial translocation to the immunological synapse (61), and mtROS formation (62). When activated, Th cells proliferate and differentiate into pro- or anti-inflammatory Th cells [T regulator cells (Treg)] (63, 64). These two antagonistic differentiations require distinct metabolic programs. Proinflammatory Th17 cells are highly glycolytic while Treg, in contrast, have high lipid oxidation rates. *In vitro*, etomoxir, an inhibitor of carnitine palmitoyltransferase-1 on the outer face of the inner mitochondrial membrane, impairs differentiation into Treg, but does not affect differentiation into proinflammatory Th cells (63). Pyruvate dehydrogenase (PDH) activity, the enzyme that positively links glycolysis and Krebs cycle in the mitochondria, is reduced in Th17 cells by PDH kinase 1 (PDHK1), and inhibition or knockdown of PDHK1 selectively suppresses Th17 cells and increases Tregs. Moreover, in mice, inhibition of PDHK1 leads to decreased Th17 cells, increased Treg, and protection against experimental autoimmune encephalomyelitis and colitis (64).

## Conclusion

Mitochondria are central hubs of inflammation regulation. Knowing that inadequate inflammation is involved in a wide range of diseases, the emerging understanding of how mitochondria fine tune the inflammation might open broad therapeutic avenues.

## Author Contributions

AM, LB, AC, GA, JP, JS, and BG have contributed to the conception or design of the work; acquisition, analysis, and interpretation of data for the work; drafted the work or revising it critically for important intellectual content; and approved of the version to be published.

## Conflict of Interest Statement

The authors declare that the research was conducted in the absence of any commercial or financial relationships that could be construed as a potential conflict of interest.
